# Carbon Isotopic Evidence for Gas Hydrate Release and Its Significance on Seasonal Wetland Methane Emission in the Muli Permafrost of the Qinghai-Tibet Plateau

**DOI:** 10.3390/ijerph19042437

**Published:** 2022-02-20

**Authors:** Xiaoqian Li, Jianwei Xing, Shouji Pang, Youhai Zhu, Shuai Zhang, Rui Xiao, Cheng Lu

**Affiliations:** 1 The Key Laboratory of Unconventional Oil & Gas Geology, China Geological Survey, Beijing 100083, China; lixiaoqian@cug.edu.cn (X.L.); zhuyouhai@mail.cgs.gov.cn (Y.Z.); zhangshuai870517@163.com (S.Z.); didaxr@163.com (R.X.); jaluch@126.com (C.L.); 2 School of Environmental Studies, China University of Geosciences, Wuhan 430074, China; 1201910330@cug.edu.cn; 3 Oil and Gas Survey, China Geological Survey, Beijing 100083, China

**Keywords:** carbon isotopic signature, gas hydrate, wetland methane emission, permafrost, Qinghai-Tibet Plateau

## Abstract

In order to determine the significant role of gas hydrate in seasonal wetland methane emission at the drilling-affected permafrost, the carbon isotopic monthly field monitoring of methane (CH_4_), as well as carbon dioxide (CO_2_), emitted from near-surface soil and a gas hydrate drilling well (DK-8) was conducted in the Muli permafrost of the Qinghai-Tibet Plateau. The methane source effused from the well DK-8 was calculated as −25.9 ± 1.4‰ and −26.5 ± 0.5‰, respectively, by the Keeling and Miller Tans plots, with the carbon isotope fractionation (ε_C_) between CO_2_ and CH_4_ from −25.3‰ to −32.1‰. The carbon isotopic signatures are indicative of thermogenic origin associated with gas hydrate dissociation. The near-surface soil-emitted methane has δ^13^C_CH4_ values between −52.0 ± 1.2‰ and −43.2 ± 1.8‰ with the heaviest in December and the lightest in July. Further, the ε_C_ values of near-surface soil-emitted gases were between 28.6‰ and 47.9‰, significantly correlated with the δ^13^C_CH4_ values. The linear correlation between ε_C_ and δ^13^C_CH4_ values indicated binary end-member of microbial and thermogenic sources control the seasonal variation of wetland methane emission. The thermogenically derived methane was identified as the dominant methane source in autumn and winter, compared with the increasing contribution of microbially derived methane in spring and summer. The finding provides reliable evidence for gas hydrate release on the seasonal wetland methane emission in the Muli permafrost affected by drilling activities. The combined application of ε_C_ and δ^13^C_CH4_ to distinguish thermogenic from biogenic methane is well established and powerful in complex environments, which can provide an improved constraint on source apportionment for wetland emitted methane in the permafrost of the Qinghai-Tibet Plateau.

## 1. Introduction

Methane (CH_4_) is an important greenhouse gas that provides the second-largest contribution to historical global warming, with stronger climate warming potential than carbon dioxide (CO_2_) [1]. Global atmospheric methane concentrations have increased rapidly since 2007 at a renewed growth rate of 6.9 ± 2.7 ppb yr^−1^ [2]. Wetland methane emissions, the largest natural source in the global CH_4_ budget, play an emerging role in driving 21st-century climate change [3]. The carbon pools in permafrost regions are large reservoirs susceptible to climate change and can drive positive feedback to climate warming [4,5,6,7,8]. Methane emission in the thawing permafrost is key to the greenhouse gas budget on climate-relevant timescales [9]. Therefore, the wetland methane emission process strongly associated with wetland carbon cycling in permafrost regions is of great significance to understanding climate feedback and mitigating global warming.

The Qinghai-Tibet Plateau is the largest high-altitude permafrost region on Earth, with a wetland area covering approximately 130,000 km^2^ [10]. In addition, substantial amounts of soil organic carbon [11,12,13] and thermogenic hydrocarbons in gas hydrates [14,15,16] are stored in the Qinghai-Tibet Plateau. Compared with conventional peat wetlands, the alpine wetlands emission of the Qinghai-Tibet Plateau is unique and complex under global climate warming [17], which is highly sensitive to temperature change [18,19,20], thawing of permafrost [21,22,23], and the destabilization of gas hydrates [24,25,26]. As a result, the average atmospheric methane content in the Waliguan of the Qinghai-Tibet Plateau was reported as 1931 ± 3 ppb in 2019 [27] higher than the global average content (1877 ± 2 ppb), with an annual increasing rate of 7.7 ppb during the past ten years [27] compared to the global annual increasing rate of 7.3 ppb. Source identification of wetland methane emission is an important issue for understanding the processes and mechanisms of wetland methane emission and carbon cycling in the Qinghai-Tibet Plateau.

Previous studies asserted that the source of wetland methane emission above the Muli gas hydrate reservoir in the Qilian Mountain permafrost could be hydrocarbon gases released by subterranean gas hydrates [14,26,28]. However, whether dissociation of gas hydrate when the p-T condition changes caused by natural process or anthropogenic activities has a significant impact on the wetland methane emission has not been fully constrained. The methane derived from gas hydrate release can effuse towards the near-surface soil through micro-seepage [29] and may have an impact on the surface carbon circulation system, forming a new source of methane emission from wetland soils in permafrost regions. Recent research of 42 monitoring sites over an area of 3 km^2^ in the Muli permafrost [30] reported some δ^13^C_CH4_ values of free gas in the near-surface soil were between −48.0‰ and −42.2‰, which indicated methane of thermogenic origin. The relationship between the emission by soils and effusion from gas hydrates was determined through correlation analyses of soil-adsorbed hydrocarbons [30,31]. Nevertheless, it still lacks direct and sufficient evidence for the effect of methane from gas hydrate dissociation on soil methane emission.

The distinct δ^13^C_CH4_ signatures are frequently used as a useful constraint to distinguish between thermogenic and biogenic methane sources. The thermogenic methane is generally enriched in ^13^C compared with biogenic methane that has δ^13^C_CH4_ values more negative than −50‰ and up to −100‰ [32]. However, situations, such as the mixing of different natural gases or where extreme substrate depletion and CH_4_ consumption by oxidation, could produce ambiguous methane isotope signals [32,33]. The carbon isotope separation (ε_C_) between CH_4_ and CO_2_ (defined by ε_C_≈δ^13^C_CO2_-δ^13^C_CH4_) can remain consistent [32] despite large variations in the actual δ^13^C_CH4_ values and indicative of the particular methanogenic pathway. Accordingly, the combination of δ^13^C_CH4_ with ε_C_ values may reliably identify methane sources in complex environments. 

The grid-monitoring results of near-surface soil samples at the gas hydrate reservoir region in the Muli permafrost [31] showed the intensive drilling site (DK-1,2,3,7,8,9, 12–13) was a unique area where the soil-emitted methane has lower δ^13^C_CH4_ (−65‰ to −80‰) in May whereas relatively higher δ^13^C_CH4_ (−54‰ to −40‰) in August and higher δ^13^C_CH4_ (−38‰ to −26‰) in October. Therefore, we conducted a twelve-month field monitoring of stable carbon isotopic compositions of CO_2_ and CH_4_ in the free gas of the near-surface soil at the above intensive drilling site in the Muli permafrost in 2017. The hydrocarbon gases emitted from one of the drilling wells (DK-8) were also monitored at the same time to determine their stable carbon isotopic signatures. This study is aimed to provide new evidence for gas hydrate release as a significant source of wetland methane emission in the Muli permafrost, to reveal the effect of gas hydrate release on wetland methane seasonal emission, and further, to imply the influence of anthropogenic activities, such as drilling, on wetland methane emission in the permafrost of the Qinghai-Tibet Plateau. 

## 2. Materials and Methods

### 2.1. Study Area

**Figure 1:**
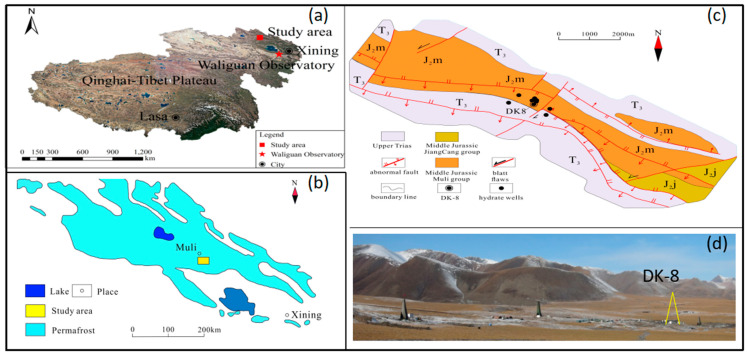
(**a**) Location of the study area in the Qinghai-Tibet Plateau, (**b**) permafrost distribution in the Qilian Mountains, (**c**) geological map showing the faults and the gas hydrate drilling wells, and (**d**) landscapes near the well DK-8.

The study area is located in the Muli permafrost of the Qilian Mountains in the northeastern margin of the Qinghai-Tibet Plateau (Figure 1a,b), with an altitude of approximately 4000–4300 m and an annual average temperature of −5.1 °C. The wetland ecosystem is dominated by alpine meadow, which is influenced by permafrost freezing and thawing driven by seasonal temperature variation. The alpine-typed permafrost covers an area of 1 × 10^5^ km^2^ in the Qilian Mountains, with a thickness regionally ranging from 60 to 95 m and an active layer of 1.5–2.0 m [34]. 

The study area is tectonically situated in the western Middle Qilian block formed during the Caledonian Movement (513–386 Ma), adjacent to the South Qilian structural zone [35], and it is also situated in the Muli depression of the south Qilian basin. The exposed strata are Quaternary, Middle Jurassic, and overturned Triassic, which thrust onto the Middle Jurassic. Large NW–SE trending thrust faults developed in the study area (Figure 1c). These thrusts or fractures may serve as paths for gas to migrate upwards from the deep layers, and some of the fractures may serve as temporary gas hydrate-bearing reservoirs [36]. 

Gas hydrate exploration and scientific drilling work have been performed in permafrost regions in the Qilian Mountains since 2000 [37,38]. A large amount of gas was released when the gas hydrate-bearing cores were extracted under air-tight conditions. Raman spectroscopy identified CH_4_ as the primary hydrate-forming gas, with secondary components of C_2_H_6_, C_3_H_8_, and CO_2_ [14]. The geological and geochemical field exploration work indicated the existence of natural gas hydrate in the target area [36,37,38,39]. The gas hydrate is a structure II gas hydrate, mainly stored in siltstone and mudstone of the Middle Jurassic Jiangcang Formation, and secondly, sandstone is unstable and discontinuous in distribution. The occurrence of gas hydrates is closely related to faults, and the burial depth is generally 133–396 m [40].

### 2.2. Field Monitoring Method

The monthly dynamic monitoring of methane in the free gas from the hydrate gas drilling well DK-8 and near-surface soil was conducted simultaneously in situ from January to December in 2017 in the study area. The monitoring frequency is once or twice a month. 

**Figure 2:**
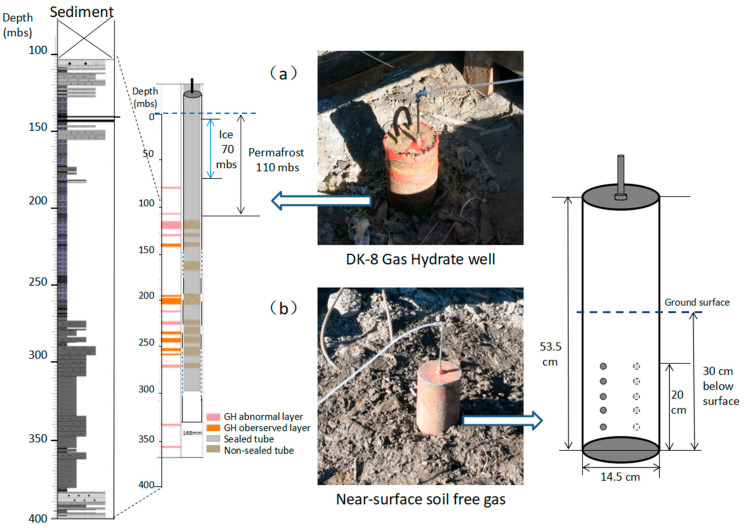
Field monitoring method, (**a**) gas hydrate drilling well DK-8, (**b**) near-surface soil-free gas monitored by the designed cylindrical soil respiration chamber.

The DK-8 well with a diameter of 16.8 cm was drilled to a depth of approximately 400 m, with the presence of gas hydrate and its associated anomalies mainly within intervals of 150–300 mbs (meter below surface), shown in Figure 2a. The core lithology is mainly composed of sandstone, siltstone, mudstone, and oily shale. The base of the permafrost is around 110 m, revealed by drilling, coexisting with underground ice around 70 mbs. The DK-8 was twice tested for exploring gas hydrates in 2011 and 2016, respectively. During the monitoring period, the mouth of the DK-8 was sealed, and the headspace gas can be only derived from the gas emission of the 150–300 mbs layers. 

The free gas from the near-surface soil was monitored using a detachable cylindrical soil respiration chamber that was 14.5 cm in diameter and 53.5 cm in height (Figure 2b). The cylindrical chamber was embedded in a pre-excavated hole with a depth of 30 cm near the well DK-8. After sealing the chamber in the hole, the whole system was circulated with the surrounding soil only through the several sieve holes in the chamber wall 10–30 cm blew the ground. Disturbance from the mixing of air and soil gas through the soil surface was assumed to be negligible because of the fine texture and compact nature of the soil and gas-tight measurement chamber. 

### 2.3. Measurements and Data Processing

The sampling ports from the well DK-8 and the soil chamber were connected, respectively, to different gas inlets of a portable Picarro isotope analyzer (G2201-i; Cavity Ring-Down Spectroscopy, Picarro, USA). On all sampling occasions, the contents (ppm) and carbon isotope composition (δ^13^C, ‰ vs. PDB) of CH_4_ together with CO_2_ were simultaneously measured in situ using the Picarro G2201-i isotope analyzer. The Picarro G2201-i was checked regularly against working standards, and adjustments were made before each field monitoring. The working standards used were a CH_4_ standard gas of 2 ppm and 10 ppm with the δ^13^C gradients of 0‰, −20‰, −40‰, and −65‰, and used CO_2_ standard gas of 300 ppm and 600 ppm with the δ^13^C gradient of 0‰, −10‰, −20‰, and −30‰, respectively. These standard gases were calibrated by the National Institute of Metrology, China. 

The readings were collected under a high-accuracy CH_4_-CO_2_ compound mode at intervals of 5 min for a total duration of at least 4 h per monitoring. The performance drift of the instrument was checked during the continuous determination for 24 h. The content precision (5min, 1σ) is 5 ppb (+0.05% of reading) for CH_4_ and 200 ppb (+0.05% of reading) for CO_2_, with the guaranteed δ^13^C precision (5min, 1σ) <1.0‰ for CH_4_ and <0.2‰ for CO_2._ Moreover, the instrument itself has a temperature control function with an accuracy of 0.5%. The measurement can only be started when the temperature of the instrument chamber reaches 45 ℃, thus avoiding the interference of ambient temperature change. 

The two gas inlets were alternately switched and measured in succession for each sampling and measurement. To avoid physical fluctuations caused by switching gas inlets, only the measured data during the intermediate 3-min stationary period were taken as valid data to calculate. All valid readings of the total duration per monitoring are calculated as mean ± standard error and presented as the final result for each measurement. The calculated measurements of CH_4_ and CO_2_ content, δ^13^C_CH4_ and δ^13^C_CO2_, as well as the calculated carbon isotope fractionation ε_C_ between CO_2_ and CH_4_ (ε_C_ = δ^13^C_CO2_ − δ^13^C_CH4_) for the free gas of the DK-8 hydrate gas drilling well and the active soil layer are shown in Table 1. 

## 3. Results

### 3.1. Monthly Temperature Variation of the Soil upper Active Layer

**Figure 3:**
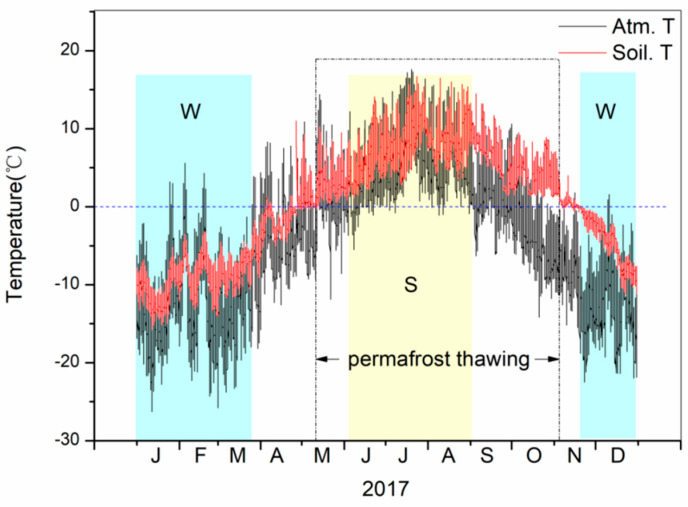
Daily temperature curves of low-level air and soil active layer in the Muli permafrost of the Qilian Mountains from January to December in 2017.

The monthly temperature variation of the soil’s upper active layer, as well as the low-level air, at the gas hydrate drilling area in the Muli permafrost is shown in Figure 3, based on the average daily monitoring data in 2017. The soil temperature varied with the atmospheric temperature in a range of −16–16 °C, lower than that of the atmospheric temperature (−25–18 °C). The soil temperature was observed above 0 °C first in early April and was always greater than 0 °C between May and October, which is the main period of permafrost thawing. The frozen period characterized by the soil temperature lower than 0 °C was from November to next April. 

### 3.2. Methane Content and Carbon Isotopic Compositions Derived from the Drilling Well DK-8

**Figure 4:**
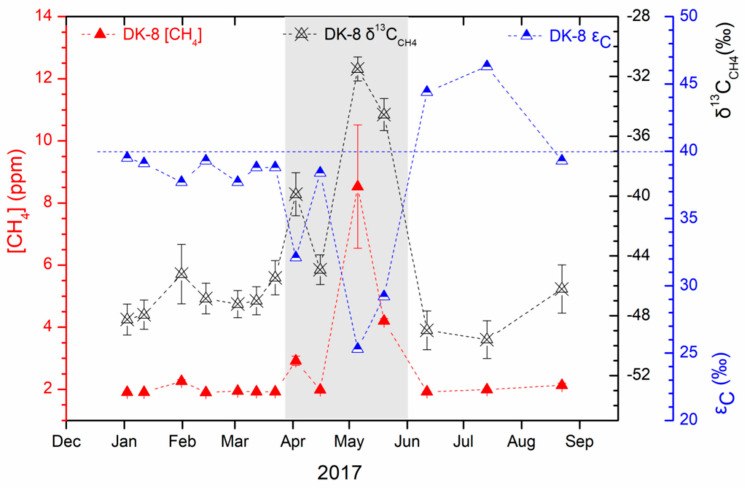
Content and stable carbon isotopic composition (δ^13^C_CH4_) of methane and carbon isotope separation (ε_C_) between CH_4_ and CO_2_ effused from the gas hydrate drilling well DK-8.

The monthly variation in content and carbon isotopic composition of methane derived from the hydrate drilling well DK-8 during the period from January to August in 2017 is shown in Figure 4. The measured methane contents showed a large variation from 1.904 ± 0.001 to 8.530 ± 1.983 ppm. The methane was constant at a low concentration of 1.967 ± 0.130 ppm from January to March and of 2.015 ± 0.108 ppm from June to August, whereas it significantly increased to 2.920 ppm in early April and soared to 8.530 ppm in early May and 4.201 ppm in late May. The measured δ^13^C_CH4_ values varied between −49.6‰ and −34.6‰, parallel with methane contents (Figure 4). Overall, the higher CH_4_ content had less negative δ^13^C_CH4_, while lower CH_4_ content had more negative δ^13^C_CH4_. Specifically, for the higher content of CH_4_, the δ^13^C_CH4_ values were −39.9‰ in early April, −31.5‰ in early May, and −34.6‰ in late May, compared with −47.1 ± 1.5‰ for the steady low content of CH_4_. The carbon isotope fractionation (ε_C_) between CO_2_ and CH_4_ varied between 25.3‰ and 46.3‰, with the highest in July and the lowest in May. The ε_C_ values are negatively correlated with the δ^13^C_CH4_ values with a Pearson’s r of −0.94997. 

### 3.3. Methane Content and Carbon Isotopic Compositions of Free Gas from the Upper of Soil Active Layer

**Figure 5:**
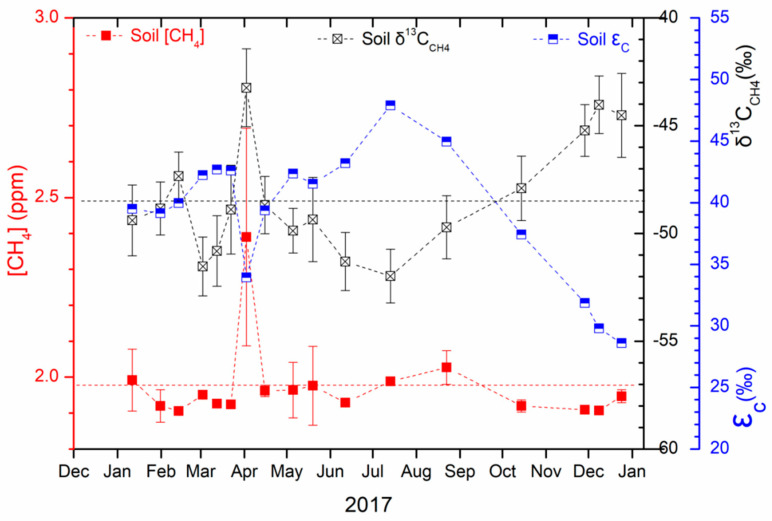
Content and stable carbon isotopic composition of methane (δ^13^C_CH4_) and carbon isotope fractionation (ε_C_) between CO_2_ and CH_4_ in the free gas of the soil active layer.

The CH_4_ content of the soil chamber varied from 1.906 ± 0.003 to 2.390 ± 0.303 ppm in 2017, with an average of 1.973 ± 0.113 ppm. Their δ^13^C_CH4_ values, representing that of the upper soil active layer, ranged between −52.0 ± 1.2‰ and −43.2 ± 1.8‰ with an average of −48.4 ± 2.7‰. The δ^13^C_CH4_ values measured in March–August (−50.2 ± 1.1‰) were lower than those measured in October–February (−46.7 ± 2.1‰). Particularly, the CH_4_ with soaring content (2.390 ± 0.303 ppm) in early April had significantly higher δ^13^C_CH4_ values (−43.2 ± 1.8‰) than that in other months (Figure 5), suggesting a significant increase of the methane source that has more positive δ^13^C_CH4_. The carbon isotope fractionation (ε_C_) between CO_2_ and CH_4_ ranged between 28.6‰ and 47.9‰, with the highest in July and the lowest in December. The significant increasing and decreasing trend of the ε_C_ values were observed in the periods from April to July and from July to December, respectively. The ε_C_ values were negatively correlated with the δ^13^C_CH4_ values with a Pearson’s r of −0.9115. 

## 4. Discussion

### 4.1. Carbon Isotopic Signatures for Methane Derived from Gas Hydrate Dissociation

**Figure 6:**
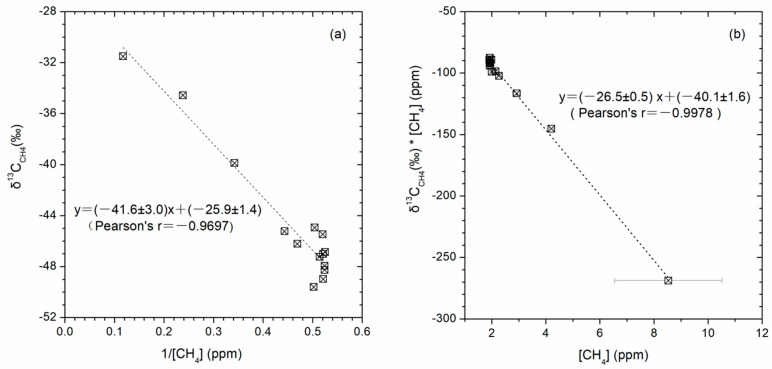
Qualitative assessment of source CH_4_ isotopic composition. (**a**) Keeling plot showing the relationship between δ^13^C_CH4_ and the inverse of CH_4_ content with the intercept of the regression as δs. (**b**) Miller-Tans plot showing the relationship between δ^13^C_CH4c_* [CH_4_] content and the slope of the regression as δs.

We used Keeling and Miller Tans plots [41,42,43] to explore the potential source isotopic signature of fresh CH_4_ and CO_2_ escaping from the drilling well DK-8. The source isotopic signature for CH_4_ (δ_s_) was calculated as the intercept value (−25.9 ± 1.4‰) of the regression associated with the Keeling plot (Figure 6a) and as the slop value (−26.5 ± 0.5‰) of the regression associated with the Miller Tans plot (Figure 6b), respectively. There was no clear distinction in identified δ_s_ values between the two methods. The less negative δ^13^C_CH4_ values are typical of thermogenically-derived methane sources enriched in ^13^C (−50‰ to −20‰, [32]). The isotopic signature of the CH_4_ source for the well DK-8 is also consistent with δ^13^C_CH4_ values (−17.7‰ to −34.7‰) of headspace gases from gas-hydrate-bearing DK-8 drill cores at depths of 184–291m [36]. Gases from the gas hydrate samples in the Muli permafrost were mainly thermogenic, either of coal-derived or oil-derived origin, discerned by the combined plots of δ^13^C_1_-C_1_/(C_2_ + C_3_) [30,40,44,45,46], δ^13^C_1_-δ^13^C_2_-δ^13^C_3_ [47,48], and δ^13^C_1_-δD_1_ [44]. However, the Pearson’s correlation coefficients between δ^13^C_C__O2_ and 1/[CO_2_] and between δ^13^C_C__O2_*[CO_2_] and CO_2_ content were low (r = 0.1768 and r = −0.5690, *p* > 0.05, respectively). Thus, the traditional “Keeling plot” or “Miller Tans plots” two end-member mixing model cannot estimate the fresh CO_2_ source. The CO_2_ contents (422.0 ± 37.7 ppm) were considerably greater than the CH_4_ contents (1.98 ± 0.11 ppm), suggesting the importance of some other mechanisms of CO_2_ production. Therefore, besides the hydrate-forming gas, the CO_2_ source is not specified in this study, which may come from the deep depth or from soil organic decomposition.

The increasing CH_4_ content of well DK-8 in April–May was observed with less negative δ^13^C_CH4_ values than −40‰ (Figure 4), indicating a substantial proportion of releasing CH_4_ from gas hydrate dissociation. The CH_4_ from gas hydrate dissociation in the permafrost layer at a depth of 150−305 m can diffuse into well DK-8 and be trapped and accumulated in the ice of the well during upward diffusion when the soil temperature is lower than 0 °C. The occurring of a considerable increase of CH_4_ content of the well DK-8 coincides with the initial melting of the ice of the well in early April when the soil temperature first raises above 0 °C. The peak of the CH_4_ content also coincides with the complete melting process in early May when the soil temperature is always above 0 °C. 

**Figure 7:**
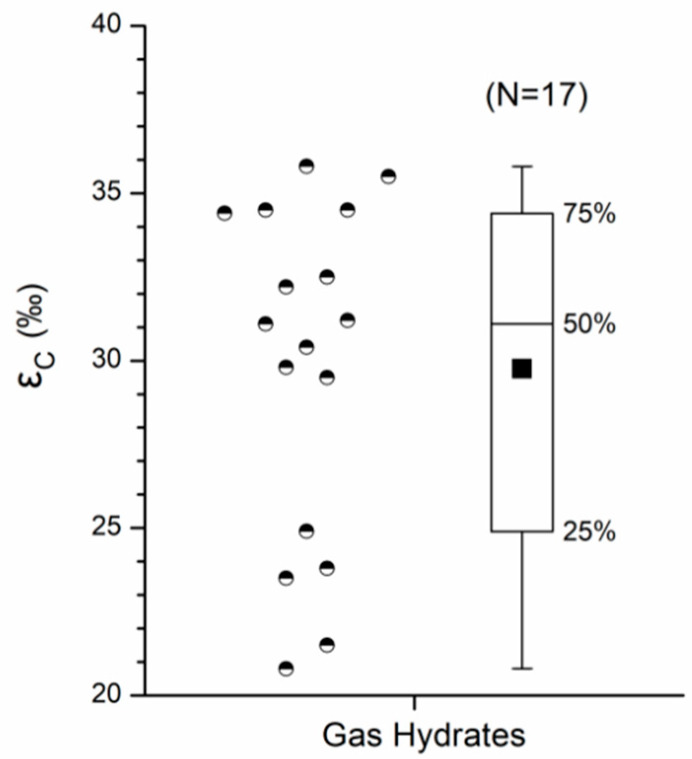
The boxplot of the calculated ε_C_ values of gas hydrates in the Muli permafrost.

The carbon isotope fractionation (ε_C_) between CO_2_ and CH_4_ can be indicative of the particular methanogenic pathway [32]. The ε_C_ values of gas hydrates in the Muli permafrost are calculated based on the reported δ^13^C data of gas components (CH_4_ and CO_2_) of the drilled gas hydrate samples summarized by Dai et al. [48]. The ε_C_ values are characterized by 21.5–35.8‰, with an average of 29.8‰ (Figure 7), associated with the average δ^13^C_CH4_ and δ^13^C_CO2_ of −45.3 ± 5.8‰ and −13.2 ± 7.3‰. The calculated ε_C_ values of gases from well DK-8 that correspond to more CH_4_ content and higher δ^13^C_CH4_ were 25.3–32.1‰, which is consistent with the thermogenic gas hydrate dissociation. This consistency shows no to little carbon isotopic fractionation during upward transport through the drilling wells. Moreover, the ε_C_ values of gas hydrates are distinctly lower than those associated with microbial methanogenesis predominantly by carbonate reduction (ε_C_ from 49‰ to over 100‰, [32]) or by fermentation of methylated substrates (ε_C_ typically between 40‰ and 55‰, [32]) and distinctly higher than those associated with microbial methane oxidation (ε_C_ generally less than 10‰, [32]). Therefore, the ε_C_ of 20–36‰ can be a carbon isotopic signature of thermogenic gas hydrate. The lower ε_C_ values associated with the δ^13^C_CH4_ higher than −40‰ further suggest considerable contribution from gas hydrate in April–May. The higher ε_C_ values (44.4–46.3‰) associated with lower δ^13^C_CH4_ (~−49‰) indicate an increasing contribution of a microbial source in June–July due to the elevated temperatures.

### 4.2. Sources of Seasonal Wetland Methane Emission Affected by Gas Hydrate Drilling 

The flux and area-weighted mean global source signature from wetlands is −62‰ [49], which is similar to the mean microbial signature of 61.5 ± 0.6‰ [50]. The source signature map resolves a latitudinal difference of ~10‰ between northern high-latitude (mean 67.8‰) and tropical (mean 56.7‰) wetlands [49]. However, the annual average of δ^13^C_CH4_ in the study area is −48.4 ± 2.7‰, significantly more enriched than the global source isotopic signature. The soil-emitted δ^13^C_CH4_ in summer (−51.3 ± 0.9‰) of the study area is similar to that in two bogs (−53.0 ± 5.5‰ and −55.3 ± 5.5‰) separated by >1000 km over the Qinghai-Tibetan Plateau [51], but distinctly different from that of the northern European wetlands (−71 ± 1‰) [52]. In particular, more enriched δ^13^C_CH4_ of the soil-emitted methane was observed in December than in July–August, reflecting highly variable seasonal wetland methane sources. Compared with wetlands away from gas hydrate in the Qinghai-Tibetan Plateau, there was no significant difference of soil-emitted δ^13^C_CH4_ between summer (from −60.6‰ to −54.6‰) and winter (−65.5‰ to −55.8‰) [53].

**Figure 8:**
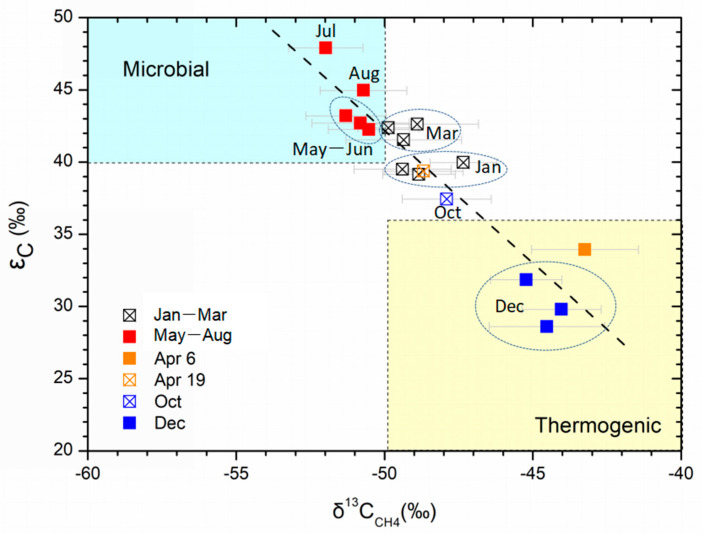
Carbon isotopic composition of wetland methane emission showing the seasonal variation of soil-emitted methane source derived from microbial and thermogenic origins.

The gases of thermogenic gas hydrates samples in the Muli permafrost of the Qilian Mountains had distinctive δ^13^C_CH4_ values heavier than −50‰ and ε_C_ values lower at 20–36‰ from the microbially-derived methane with δ^13^C_CH4_ lighter than −50‰ and ε_C_ values more than 40‰. Therefore, the isotope signatures of δ^13^C_CH4_ combined with ε_C_ can be adequate to reliably distinguish microbially or thermogenically derived methane. The two dominated microbial and thermogenic sources controlling seasonal variation of the wetland methane emission in the study area are indicated by linear correlation of binary mixing in the diagram of ε_C_ versus δ^13^C_CH4_ (Figure 8). The samples collected in spring and summer plotted in the microbial zone indicated the soil-emitted CH_4_ being dominated by fermentation of methylated substrates (δ^13^C_CH4__=_ −50‰ to −60‰, ε_C_ = 40–60‰, [32]). Further, the samples in autumn and winter are plotted beyond the microbial zone and along the fitting line into the thermogenic zone, indicating an increasing contribution of thermogenic gas hydrates. A response of soil methane emission to the CH_4_ effusion peak out of well DK-8 occurred on April 6 and can be observed by the CH_4_ surge (2390 ppm), less negative δ^13^C_CH4_ (−43.2‰), and lower ε_C_ (33.9‰) during the same period. The soil-emitted CH_4_ on April 6 plotted in the thermogenic zone (Figure 8) suggests the accumulated thermogenic gas hydrate dissociation in the ice of drilling wells is an important source of soil-emitted methane.

The combined effects of both microbially and thermogenically derived methane control the seasonal variation of wetland methane emission. This finding further suggests gas hydrate dissociation affected by drilling activities is an important source of methane emission from wetlands in the Muli permafrost. During spring and summer, microbes are the dominant source of soil-emitted methane, although the thermogenic methane emission from subterranean gas hydrates can be accelerated through new channels resulting from permafrost thawing. The increase in temperature accompanied by permafrost thawing promoted an exponential boost in the scale of microbial communities and activity [54,55,56] during the transition from spring to summer, which results in increasing methane produced by methanogenic microbes from May to July. However, with an increase in temperature to a certain extent, the upper limit of the environmental carrying capacity of methanogenic microbial communities is reached [57], which leads to a lower contribution of microbial methane in August than in July.

During the transition from late summer to autumn, microbial activities become weaker as the temperature decreases and the consequential reduction in methane production by methanogens. As a result, the methane contribution from the microbial source decreased, and that from the thermogenic source relatively increased in October. During winter, significant decreases occur on the scale of microbial communities and microbial activity [58], especially at temperatures below −10 °C. This leads to a substantial reduction in the amount of methane produced by methanogens. Thus, the contribution of the thermogenically derived methane relatively increased. The carbon isotope compositions of δ^13^C_CH4_ and ε_C_ of wetland emission consistently reflected the dynamic variation of relative contributions of microbial and thermogenic methane during autumn and winter. In addition, as the average daily temperature falls below zero, freezing occurs in the permafrost, which leads to the decreased effusion of methane from subterranean gas hydrates. The dominance of thermogenically derived methane in December is shown in Figure 8, suggesting that the subterranean gas hydrates dissociation may seep through faults or drainage systems or drilled-through permafrost layers upward into the wetland.

## 5. Conclusions

The twelve-month systematic field monitoring of methane sources at a typical gas hydrate drilling site in the Muli permafrost of the Qilian Mountains provided carbon isotopic evidence that thermogenic gas hydrate dissociation is an important source of wetland methane emission affected by drilling activities. The combined application of carbon isotope fractionation (ε_C_) between CH_4_ and CO_2_ and carbon isotopic composition of methane (δ^13^C_CH4_) to distinguish thermogenic from microbial methane is well established and appears to be useful in complex environments. The carbon isotopic signatures for methane derived from gas hydrate dissociation determined by the gas hydrate drilling DK-8 is distinctly characterized by heavier δ^13^C_CH4_ of −26.5 ± 0.5‰ and lower ε_C_ of −25.3‰ to −32.1‰. The seasonal variation of methane sources of near-surface soil fluxes at the drilling site confirmed the significance of methane emission from the thermogenic gas hydrates on local permafrost wetland methane emission, especially in autumn and winter. The effect of gas hydrate dissociation on methane emission of the permafrost wetland may become more significant with the further exploration of gas hydrate resources. The effect of gas hydrate on regional wetland methane emission in the Muli permafrost requires more research to fully understand, which will allow for exploration of the mechanisms of soil methane emissions in the permafrost wetlands of the Qinghai-Tibet Plateau as the global climate warms.

## Figures and Tables

**Table 1:** Methane content and carbon isotopic compositions of methane and carbon dioxide in the gas effused from the DK8 hydrate gas drilling well and in the free gas of the soil active layer.

Sampling Date	[CH_4_] (ppm)	δ^13^C_CH4_ (‰ vs. PDB)	[CO_2_] (ppm)	δ^13^C_CO2_ (‰ vs. PDB)	ε_C_ (=δ^13^C_CO2_ − δ^13^C_CH4_) (‰ vs. PDB)
**DK8 well gas**					
2017/1/6	1.908 ± 0.018	−48.3 ± 1.0	413.6 ± 1.3	−8.8 ± 0.3	39.5
2017/1/15	1.909 ± 0.001	−47.9 ± 1.0	415.9 ± 5.4	−8.9 ± 0.3	39.1
2017/2/4	2.259 ± 0.058	−45.2 ± 2.0	331.4 ± 9.8	−7.5 ± 0.6	37.7
2017/2/17	1.904 ± 0.001	−46.9 ± 1.0	435.8 ± 26.5	−7.5 ± 1.0	39.3
2017/3/6	1.944 ± 0.006	−47.2 ± 0.9	463.6 ± 8.3	−9.5 ± 0.5	37.7
2017/3/16	1.921 ± 0.005	−47.0 ± 0.9	434.2 ± 14.6	−8.2 ± 0.7	38.8
2017/3/26	1.923 ± 0.004	−45.5 ± 1.1	462.7 ± 6.9	−6.6 ± 1.2	38.8
2017/4/6	2.920 ± 0.148	−39.9 ± 1.4	337.9 ± 10.0	−7.7 ± 0.6	32.1
2017/4/19	1.983 ± 0.007	−44.9 ± 1.0	455.1 ± 14.0	−6.5 ± 1.0	38.4
2017/5/9	8.530 ± 1.983	−31.5 ± 0.8	302.6 ± 22.2	−6.2 ± 0.9	25.3
2017/5/23	4.201 ± 0.078	−34.6 ± 1.1	325.9 ± 1.5	−5.4 ± 1.2	29.2
2017/6/15	1.920 ± 0.022	−49.0 ± 1.3	405.6 ± 0.8	−4.6 ± 1.2	44.4
2017/7/17	1.993 ± 0.007	−49.6 ± 1.3	429.6 ± 2.4	−3.3 ± 0.3	46.3
2017/8/26	2.132 ± 0.069	−46.2 ± 1.6	395.0 ± 5.5	−6.9 ± 0.9	39.3
**Free gas of the active soil layer (10–30 cm depth)**					
2017/1/15	1.992 ± 0.086	−49.4 ± 1.6	437.9 ± 6.0	−9.9 ± 0.4	39.5
2017/2/4	1.920 ± 0.045	−48.8 ± 1.2	471.3 ± 36.0	−9.7 ± 2.8	39.2
2017/2/17	1.906 ± 0.003	−47.3 ± 1.2	435.4 ± 23.6	−7.4 ± 0.9	40.0
2017/3/6	1.951 ± 0.006	−50.5 ± 1.4	464.2 ± 8.0	−9.3 ± 0.4	41.3
2017/3/16	1.926 ± 0.005	−50.8 ± 1.6	436.3 ± 14.5	−8.1 ± 0.6	42.7
2017/3/26	1.924 ± 0.069	−48.9 ± 2.1	466.9 ± 8.2	−6.3 ± 1.3	42.6
2017/4/6	2.390 ± 0.303	−43.2 ± 1.8	474.6 ± 21.9	−9.3 ± 0.7	33.9
2017/4/19	1.962 ± 0.016	−48.7 ± 1.3	445.8 ± 23.0	−9.3 ± 0.4	39.4
2017/5/9	1.964 ± 0.077	−49.9 ± 1.0	426.5 ± 14.0	−7.5 ± 0.8	42.4
2017/5/23	1.976 ± 0.110	−49.4 ± 2.0	407.6 ± 1.8	−7.8 ± 0.9	41.5
2017/6/15	1.929 ± 0.012	−51.3 ± 1.4	438.4 ± 12.0	−8.1 ± 0.9	43.2
2017/7/17	1.988 ± 0.005	−52.0 ± 1.2	503.5 ± 4.1	−4.1 ± 0.3	47.9
2017/8/26	2.027 ± 0.047	−50.7 ± 1.5	441.8 ± 28.6	−4.8 ± 0.8	46.0
2017/10/18	1.920 ± 0.017	−47.9 ± 1.5	425.3 ± 20.7	−10.5 ± 1.5	37.4
2017/12/2	1.909 ± 0.001	−45.2 ± 1.2	778.7 ± 49.8	−13.4 ± 1.0	31.9
2017/12/12	1.907 ± 0.004	−44.0 ± 1.3	663.2 ± 12.4	−14.2 ± 1.3	29.8
2017/12/28	1.947 ± 0.018	−44.5 ± 1.9	576.4 ± 10.5	−15.9 ± 2.1	28.6

## Data Availability

The data generated or analyzed during this study were included in this manuscript.
